# Trends in attitudes towards female genital mutilation among ever-married Egyptian women, evidence from the Demographic and Health Surveys, 1995–2014: paths of change

**DOI:** 10.1186/s12939-016-0324-x

**Published:** 2016-02-24

**Authors:** Ronan Van Rossem, Dominique Meekers, Anastasia J. Gage

**Affiliations:** Department of Sociology, Universiteit Gent, Korte Meer 3-5, 9000 Ghent, Belgium; Department of Global Community Health and Behavioral Sciences, School of Public Health and Tropical Medicine, Tulane University, New Orleans, LA USA

**Keywords:** Female genital mutilation, Egypt, Attitudes

## Abstract

**Background:**

Over the past few decades Egypt has attempted to limit and control female genital mutilation (FGM). However, these efforts have not succeeded in curbing the practice, which maintains wide popular support and is firmly embedded in local traditions and structures. An attitudinal change is therefore a prerequisite for any successful campaign against FGM. This paper charts the evolution of beliefs that the practice of FGM in Egypt should be stopped.

**Method:**

This paper examines trends in opposition to FGM among ever-married women in Egypt between 1995 and 2014, using six waves of the *Egypt Demographic and Health Surveys*.

**Results:**

The results show that the percentage of ever-married women who think the practice of FGM should be stopped rose from 13.9 % in 1995 to 31.3 % in 2014. The central question here is whether this trend exists because new cohorts of young married women are more modern and more opposed to the practice, or because opposition to FGM has spread through multiple segments of society. Our results show that back in 1995 opposition to FGM was concentrated in two groups: non-circumcised women, and wealthy, highly educated urban women. Between 1995 and 2014 opposition to FGM increased considerably among other groups of women.

**Conclusion:**

Our results show that the observed increases in opposition to FGM are not caused by younger cohorts of married women who oppose FGM, nor by the expansion of the groups most likely to oppose FGM. Rather, the results imply that the belief that FGM should be stopped spread to all walks of life, although poorly educated rural women remain least likely to oppose FGM.

## Background

For several decades the Egyptian authorities have tried, to no avail, to curb and regulate FGM. In 2007 and 2008 laws were passed that banned the practice [1, 2]. However, it remains unclear how rigidly these laws are enforced, especially given the political upheavals since 2011. Although the 2007 law prohibited general practitioners from performing FGM, in a study of FGM in Upper Egypt Rasheed, Abd-Ellah and Yousef [3] found that the incidence of FGM remained very high, and that most cuttings were still performed by general practitioners.

**Fig. 1:**
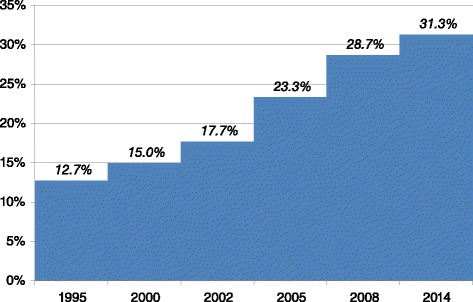
Trends in the percentage of ever-married women who believe FGM should be discontinued

Although female genital mutilation (FGM) or female circumcision is still nearly universal in Egypt, there is some evidence that the social and political climate regarding FGM is changing. According to the 2014 *Egypt Demographic and Health Survey* (EDHS) 92 % of ever married women between the ages of 15 and 49 are circumcised [4]. However, the prevalence of FGM among 20–24 year old ever married women is only 87 %, compared to about 95 % for 35 to 49 year olds. El-Gibaly et al. [5] also demonstrated that the prevalence of FGM among girls aged 10–19 is about 10 % lower than among their mothers. Other studies confirm these results [6–8], suggesting a slow decline of the practice. Given that FGM is embedded in tradition and social structure and that there is widespread support for it, the eradication of this practice – which is the objective of current legislation -- seems impossible without major changes in popular attitudes. Theories of behavior change stress the importance of attitudinal change as a necessary precursor to behavior change [9–14]. For people to abandon traditional behaviors, such behaviors must be delegitimized while alternative ones need to gain acceptance. The EDHS data indicate that opposition to FGM increased between 1995 and 2014 (see Fig. 1). In 1995 only 13 % of ever married women believed the practice of FGM should be stopped, but by 2014 this had increased to 31 %. Moreover, the data suggest that opposition to FGM spread rapidly during the first decade of this century, but somewhat less rapidly in recent years [4, 15–19].

FGM is not a harmless tradition, it brings with it serious health risks. In addition to health risks at the time of the event, such as pain, bleeding, infections, psychological trauma and even death, FGM is associated with health problems later in life, including chronic pain, infections, genital infections, sexually transmitted diseases, decreased sexual pleasure, birth complications, depression, post-traumatic stress disorder, infertility, etc. As FGM has no medical usefulness, is harmful to a woman’s health, and is imposed upon her, international organizations consider it a violation of women’s rights and a manifestation of gender discrimination [20]. Although the communities where FGM is practiced may consider it a traditional custom that assures the social status of daughters, it creates inequities in health based on sex and social status.

FGM is linked to the position of women in society [20–24] and there is substantial social pressure to conform to the norm that states that FGM is a normal aspect of every woman’s life. Theories that focus on the more sociological and social psychological aspects of behavior change, such as social convention theory [25, 26], emphasize that attitudes and decisions are embedded in community structures. Attitudes toward FGM are influenced by individuals’ social environment, which tends to pressure and socialize people toward conformity. The extent to which individuals and families can withstand such pressures depends on the available sources of status, as well as on their exposure to other social environments and influences. Therefore, not all groups are equally likely to change their attitudes toward FGM.

Innovation studies have demonstrated that changes are more likely to be initiated by small groups of innovators. Innovators have a “willingness-to-change” that makes them sensitive and receptive to new ideas and practices [27] and that leads them to engage in more cosmopolitan social relationships [28, 29]. These cosmopolitan-type social relationships are detached from local social circles. Although this detachment may undermine their credibility and status in the local community, it also allows for the introduction of new practices into a population. Innovators often adopt such practices after exposure to external communication channels. Research on the diffusion of practices in developing nations shows that access and exposure to these external resources is dominated by economically advantaged and less traditional groups. Less advantaged and more traditional segments of society are not only less exposed to sources of innovation but are also ill-positioned to take advantage of them. In this light, the diffusion of attitudes and practices has an inherent role in the reproduction of status inequalities.

In such context the two groups most likely to introduce new (and possibly deviant) attitudes are the modernizing elites and marginalized groups. Modernization in this context refers to both structural factors that alter the social positions of the groups affected by it and to the emergence of new cultural patterns. Among the structural factors education and employment in occupations that require education stand out. The modern cultural pattern is characterized by rationalization, individualization, and detraditionalization. Modernizing elites not only possess alternative sources of status, but they are also exposed to new ideas and attitudes through education and through access to local, national, and international media. A number of other studies have found that highly educated and urban women are more likely believe that FGM should be discontinued [30], and that wealthier women and better educated women are less likely to intend to have their daughters cut [8, 31, 32]. Given the higher status of these groups, they may serve as role models for other segments of society. Several theoretical models, including social cognitive theory [10], social convention theory [26], and diffusion of innovation theory [33], note the importance of role models for behavior change. Role models not only affect behavior through direct contact, but also through their visibility and status in the community. Marginal groups lack the status to fulfill this role.

This paper examines whether the observed increase in opposition to FGM can be attributed completely to the fact that recent cohorts of young married women are more modernized, i.e., urban, better educated and employed in modern occupations, and therefore more likely to oppose FGM, or whether the opposition to FGM is also spreading from these modernized segments of society to the more traditional ones, which would imply that opposition to FGM is spreading to women of all walks of life.

## Data and methods

### Sample

For this study we analyze secondary data from six waves of the *Egypt Demographic and Health Survey* (EDHS): 1995, 2000, 2003, 2005, 2008 and 2014. The EDHS surveys contain data on large nationally representative samples of ever-married women. The survey questions cover topics such as fertility, contraceptive use, infant and child mortality, maternal and child health, immunization, nutrition, as well as health-related knowledge and attitudes. The surveys also include information about FGM. The pooled dataset contains information on a total of 97,274 ever-married women between the ages of 15 and 49 years. All ever-married women between 15 and 49 years who were present in the household the night before the interview were eligible to participate in the survey. All survey waves were conducted using a two-stage stratified random sample. For more detailed information on the sampling and data collection we refer the reader to the EDHS reports [4, 15–19]. The procedures and questionnaires of all DHS surveys have been reviewed and approved by the *ICF International* institutional review board (IRB) and comply with the *U.S. Department of Health and Human Services* regulations for the protection of human subjects (45 CFR 46); they have also been reviewed by an Egyptian IRB to assure compliance with Egyptian rules and laws [34]. The de-identified EDHS data are publicly available to all researchers.

### Variables

The EDHS questionnaires ask respondents whether they believe that the practice of FGM should be continued or stopped (continued, stopped, other, or do not know).^1^ The dependent variable in this study is a dichotomous variable that equals one for respondents who believe that the practice of FGM should be stopped, and zero for all other respondents. This approach provides a conservative estimate of the number of respondents who believe that FGM should be stopped.

The time effect is captured by the year of the survey. Rather than include the age of the respondent, her birth cohort (1945–49 through 1995–1999) is included. This allows us to check whether recent cohorts are responsible for the observed changes in opposition to FGM.

Three variables are used to capture the degree to which FGM attitudes may be entrenched in social, cultural and religious traditions: whether the respondent herself has been cut, religion, and the number of children ever born. Religion is coded as Muslim, Christian, other, and missing. Note that the 2000 and 2003 surveys do not include a question on religion. Hence, information on religion is missing for those two survey waves. The religious composition of the sample remained fairly stable up to 2008, with about 5 % Christians and 95 % Muslims. The 2014 survey shows a small decline in the percentage of Christian women, to below 4 %. The mean number of children ever born in the pooled sample is 3.1 children, but it declined significantly declines from a 3.7 in the 1995 EDHS to 2.7 in 2014. The overwhelming majority of the respondents (95.5 % for all survey waves combined) report that they have been circumcised. Nevertheless a small but significant increase in the percentage of non-circumcised respondents is observed, from 3.0 % in 1995 to 7.7 % in 2014.

The socio-economic background of the respondent is measured by her education level (no formal education, primary, secondary, or higher education), literacy level (illiterate, partly literate, fully literate), her occupation, and household wealth. Because of the need to keep the wealth indicator compatible across all five survey waves, we use an indicator of absolute wealth. Our wealth indicator is defined as a count of up to eight items that the respondent’s household may possess (drinking water in residence, flush toilet, finished floor, electricity, radio, television, refrigerator and bicycle).^2^ A Mokken scale analysis of these items shows that they form an acceptable scale in which the various items capture different levels of ‘difficulty’ (i.e., wealth) (Loevinger H = 0.39). On average, respondents became significantly wealthier between 1995 and 2008, after which wealth levels decreased again. The mean score on the household wealth index increased from 5.5 in 1995 to 6.3 in 2008, but declined again to 6.0 in 2014 (*p* < 0.001). For the analyses, the household wealth index was recoded in three categories: low (0–4 assets), middle (5–6 assets) and high (7–8 assets). During the period, from 1995–2014 the education level of the respondents also increased significantly. The percentage of respondents who had no formal education decreased from 43.7 % in 1995 to 24.0 % in 2014. Similarly, the percentage of fully literate respondents increased from 36.3 to 68.4 %. Despite these improvements in female education, the large majority of women were not working (81.1 % over all waves, with a low of 77.3 % in 2003 and a high of 83.9 % in 2014).

Region of residence and the degree of urbanization are also included in the analysis. The urbanization variable distinguishes between the capital and other large cities, cities, towns and the countryside.^3^ More than half of respondents (58.8 %) live in the countryside. The percentage of urban respondents fluctuates somewhat across the various survey waves, but no clear trend can be discerned.

### Statistical analyses

To correct for the stratified nature of the survey samples, sampling weights are used in all analyses. Two series of analyses of variance are conducted. For each survey wave, the first analysis of variance tests whether a predictor variable has an effect on the belief that FGM should be stopped. Specifically, the test compares the percentage of respondents who believe the practice should be stopped across categories of the predictor variable. The second series of analyses of variance tests whether the proportion of ever-married women who believe FGM should be stopped changed over time; separate tests are conducted for each category of the predictor variables. For the pairwise comparisons Tukey HSD tests are used. All reported results are significant at α = 5 %.

Next, we test whether the observed increases in opposition to FGM actually reflect a diffusion process through all segments of Egyptian society, rather than socio-demographic changes in the composition of the society, such as increased education and urbanization. To that effect, we use logistic regression analysis to calculate the relative odds of opposing FGM for each survey compared to the 1995 odds (e.g. the relative odds of opposing FGM in 2014 compared to 1995). We compare the unadjusted (zero-order) odds ratios (OR) with the adjusted (partial) odds ratios that control for the socio-demographic status variables included in the analysis. We estimate two different sets of partial ORs. The first uses the pooled data and therefore assumes that the effects of the control variables remains constant across all survey waves. The second set of partial ORs are estimated using separate equations for each wave, which allows the effects of the control variables to vary by survey year.

Discriminant analysis is used to summarize the differences in socio-demographic characteristics between those who believe the practice of FGM should be stopped and those who do not, and to assess how this changed between 1995 and 2014. Discriminant analysis is a technique that identifies how a priori defined groups, − in this case supporters and opponents of FGM in each EDHS wave -- differ in a multivariate way on a set of observed variables. It estimates linear discriminant functions (LDF) of these observed variables that maximally account for the observed multivariate inter-group variation on these variables [35, 36]. The main purpose of our discriminant analysis is to test whether or not the supporters and opponents of FGM become more similar over time. In our analysis, we define twelve mutually exclusive groups based on the combination of attitude towards FGM (FGM should be stopped vs. all others) and the six EDHS survey waves. As discriminating variables we include: region, urbanization, education level, occupation, literacy, religion, wealth, number of children, and whether the respondent herself is circumcised or not. We do not include cohort and religion in the discriminant analysis because not all cohorts and religions are present or recorded in all waves.^4^ Because the 2014 survey does not include a detailed breakdown of the level of urbanization, urbanization is dichotomized as urban vs. rural for the discriminant analysis.

## Results

### The early opponents of FGM

In 1995 only 12.7 % of ever-married women believed FGM should be stopped (1882 out of 14767). At that time, two groups stood out for their strong opposition to FGM. The first group consists of women who were not circumcised themselves, 89.5 % of whom believed the practice should be discontinued (393/439). The second group is comprised of circumcised urban women with higher education of whom 53.2 % opposed FGM (250/470). Opposition to FGM was lowest among circumcised non-Christian women with no formal education of whom only 1.7 % believed the practice of FGM should be stopped (88/5057), and circumcised women with only primary education, partly literate and from less wealthy households (wealth index ≤ 6) of whom only 2.3 % believed FGM should be discontinued (15/663).

These findings imply that back in 1995 opposition to FGM was mainly concentrated in the modernized (“westernized”) groups in society, i.e., among urban, highly educated women, and among non-circumcised women. Although this group is of non-circumcised women is quite diverse it disproportionally consists of women who are Christian (22.1 vs. 4.8 % in the circumcised group), highly educated (58.0 vs. 4.8 %), working in professional, technical or managerial positions (30.4 vs. 5.8 %), live in the capital or large cities (63.1 vs. 23.9 %), and who are from wealthy households (wealth index ≥ 7, 87.7 vs. 39.0 %). Therefore it seems that the non-circumcised group also disproportionally consists of more modernized women, i.e., urban women with better education and more likely to be employed in ‘modern’ occupations.

### Diffusion of opposition to FGM: crossing social boundaries

Table 1 shows trends in the percentage of ever-married women who believe FGM should be discontinued for each category of the predictor variables. For each category of the predictor variables, it also shows the variance (Ε^2^) in FGM opposition explained by the trend (rightmost column in Table 1), i.e., the extent of change over time. For each survey year it also shows the variance explained by the predictor variables.

**Table 1:** Proportion of ever married women who believe FGM should be discontinued, by background characteristics and over EDHS waves

N_pd_/N %_pd_	EDHS survey (year)						Ε^2^ across survey waves
	1995	2000	2003	2005	2008	2014	
Total	1882/14769 12.7 %	2326/15558 15.0 %	1620/9154 17.7 %	4536/19461 23.3 %	4742/16524 28.7 %	6807/21756 31.3 %	
Birth cohort							
Ε^2^ across categories of variable	0.003***	0.003***	0.002	0.001***	0.002***	0.003***	
1945–49	186/1286 14.5 %						
1950–54	296/2040 14.5 %	341/2163 15.8 %	67/323 20.7 %				0.002*
1955–59	315/2405 13.1 %	354/2181 16.2 %	214/1287 16.6 %	515/2310 22.3 %	194/680 28.5 %		0.014***
1960–64	361/2644 13.7 %	428/2637 16.2 %	264/1474 17.9 %	715/2849 25.1 %	592/2266 26.1 %	100/316 31.6 %	0.018***
1965–69	359/2846 12.6 %	438/2704 16.2 %	263/1509 17.4 %	709/3154 22.5 %	670/2572 26.0 %	818/2675 30.6 %	0.023***
1970–74	285/2430 11.7 %	408/2832 14.4 %	299/1621 18.4 %	782/3206 24.4 %	724/2548 28.4 %	806/2886 27.9 %	0.025***
1975–79	79/1093 7.2 %	295/2339 12.6 %	330/1713 19.3 %	912/3762 24.2 %	914/2963 30.8 %	1103/3678 30.0 %	0.033***
1980–84	2/26 7.7 %	63/700 9.0 %	166/1098 15.1 %	723/3173 22.8 %	992/3227 30.7 %	1330/4155 32.0 %	0.025***
1985–89		0/2 0.0 %	16/127 12.6 %	180/1004 17.9 %	605/2057 29.4 %	1592/4633 34.4 %	0.016***
1990–94				1/5 20.0 %	51/211 24.2 %	922/2842 32.4 %	0.002*
1995–99						136/570 23.9 %	
Region							
Ε^2^ across categories of variable	0.076***	0.091***	0.064***	0.071***	0.073***	0.062***	
Urban Governorates	835/3312 25.2 %	988/2983 33.1 %	561/1666 33.7 %	1371/3288 41.7 %	1434/2931 48.9 %	1453/2774 52.4 %	0.039***
Lower Egypt - Urban	350/1828 19.1 %	372/1945 19.1 %	300/1179 25.4 %	787/2198 35.8 %	731/1936 37.8 %	1075/2317 46.4 %	0.051***
Lower Egypt - Rural	208/4377 4.8 %	271/4880 5.6 %	320/2922 11.0 %	1108/6208 17.8 %	1094/5682 19.3 %	2171/8342 26.0 %	0.048***
Upper Egypt - Urban	296/1578 18.8 %	396/1806 21.9 %	242/1061 22.8 %	591/2410 24.5 %	648/1789 36.2 %	901/2421 37.2 %	0.026***
Upper Egypt - Rural	149/3540 4.2 %	251/3738 6.7 %	197/2327 8.5 %	582/5140 11.3 %	727/3959 18.4 %	1116/5709 19.5 %	0.031***
Frontier Governorate	45/135 33.3 %	49/207 23.7 %		98/218 45.0 %	108/227 47.6 %	91/194 46.9 %	0.038***
Religion							
Ε^2^ across categories of variable	0.035***			0.036***	0.036***	0.037***	
Muslim	1573/13974 11.3 %			3939/18397 21.4 %	4213/15737 26.8 %	6177/20915 29.5 %	0.025***
Christian	309/792 39.0 %			595/1049 56.7 %	527/782 67.4 %	623/819 76.1 %	0.074***
Other	0/1 0.0 %					7/17 41.2 %	0.040
Missing	0/2 0.0 %	2326/15558 15.0 %	1620/9154 17.7 %	2/15 13.3 %	1/5 20.0 %	0/6 0.0 %	0.001***
Respondent circumcised?							
Ε^2^ across categories of variable	0.163***	0.076***	0.078***	0.084***	0.080***	0.090***	
No	393/439 89.5 %	304/405 75.1 %	206/256 80.5 %	663/807 82.2 %	644/732 88.0 %	1329/1675 79.3 %	0.013***
Yes	1489/14331 10.4 %	2022/15153 13.3 %	1411/8885 15.9 %	3872/18645 20.8 %	4098/15788 26.0 %	5474/20075 27.3 %	0.026***
Labor market status							
Ε^2^ across categories of variable	0.088***	0.059***	0.051***	0.038***	0.033***	0.018***	
Not working	1181/11767 10.0 %	1587/12809 12.4 %	1109/7076 15.7 %	3285/15146 21.7 %	3647/13776 26.5 %	5546/18246 30.4 %	0.035***
Prof., Tech., Manag.	432/964 44.8 %	464/1119 41.5 %	304/762 39.9 %	678/1553 43.7 %	658/1264 52.1 %	754/1589 47.5 %	0.006***
Clerical	207/660 31.4 %	205/641 32.0 %	109/274 39.8 %	240/548 43.8 %	139/314 44.3 %	139/324 42.9 %	0.014***
Sales	16/360 4.4 %	9/157 5.7 %	24/101 23.8 %	53/298 17.8 %	30/115 26.1 %	89/398 22.4 %	0.053***
Agric-self employed	0/378 0.0 %	1/59 1.7 %	7/174 4.0 %	43/598 7.2 %	8/78 10.3 %	25/221 11.3 %	0.029***
Agric-employee	4/294 1.4 %	9/415 2.2 %	24/501 4.8 %	31/506 6.1 %	37/322 11.5 %	22/344 6.4 %	0.018***
Household & domestic	6/83 7.2 %						
Services	6/42 14.3 %	18/157 11.5 %	27/100 27.0 %	152/447 34.0 %	172/398 43.2 %	146/353 41.4 %	0.045***
Skilled manual	24/209 11.5 %	21/161 13.0 %	15/157 9.6 %	43/217 19.8 %	35/110 31.8 %	72/240 30.0 %	0.046***
Unskilled manual		v	0/4 0.0 %	11/145 7.6 %	12/140 8.6 %	14/41 34.1 %	0.076***
Don’t know			0/4 0.0 %	1/4 25.0 %	2/2 100.0 %	0/2 0.0 %	0.693*
Highest education level							
Ε^2^ across categories of variable	0.201***	0.165***	0.122***	0.093***	0.106***	0.064***	
No education	174/6460 2.7 %	300/6730 4.5 %	187/3451 5.4 %	758/6736 11.3 %	716/5302 13.5 %	894/5232 17.1 %	0.033***
Primary	248/3666 6.8 %	251/2845 8.8 %	194/1559 12.4 %	437/3051 14.3 %	406/2047 19.8 %	498/2232 22.3 %	0.027***
Secondary	905/3701 24.5 %	1064/4716 22.6 %	807/3284 24.6 %	2288/7660 29.9 %	2439/7236 33.7 %	3757/11275 33.3 %	0.009***
Higher	555/943 58.9 %	711/1267 56.1 %	432/860 50.2 %	1053/2015 52.3 %	1182/1940 60.9 %	1658/3017 55.0 %	0.005***
Partner’s education level							
Ε^2^ across categories of variable	0.161***	0.128***	0.084***	0.064***	0.073***	0.039***	
No education	116/4490 2.6 %	222/4675 4.7 %	135/2231 6.1 %	540/4507 12.0 %	507/3579 14.2 %	742/3721 19.9 %	0.042***
Primary	229/4047 5.7 %	241/3390 7.1 %	210/1953 10.8 %	613/3776 16.2 %	548/2817 19.5 %	739/3130 23.6 %	0.038***
Secondary	743/4486 16.6 %	938/5405 17.4 %	722/3599 20.1 %	2022/8158 24.8 %	2332/7534 31.0 %	3588/11384 31.5 %	0.019***
Higher	792/1716 46.2 %	922/2069 44.6 %	553/1368 40.4 %	1353/2990 45.3 %	1356/2593 52.3 %	1737/3518 49.4 %	0.005***
Don’t know	2/30 6.7 %	3/19 15.8 %	0/2 0.0 %				0.025
Marital status							
Ε^2^ across categories of variable	0.001**	0.001***	0.001**	0.001***	0.002***	0.001**	
Married	1782/13700 13.0 %	2193/14369 15.3 %	1517/8441 18.0 %	4244/18174 23.4 %	4453/15394 28.9 %	6410/20456 31.3 %	0.028***
Widowed	69/746 9.2 %	74/775 9.5 %	53/442 12.0 %	140/764 18.3 %	139/671 20.7 %	176/667 26.4 %	0.030***
Divorced	31/322 9.6 %	42/307 13.7 %	42/205 20.5 %	118/394 29.9 %	128/353 36.3 %	169/460 36.7 %	0.060***
Not living together		17/108 15.7 %	8/67 11.9 %	33/128 25.8 %	23/107 21.5 %	53/174 30.5 %	0.024**
Place of residence							
Ε^2^ across categories of variable	0.071***	0.093***	0.065***	0.056***	0.069***	0.052***	
Capital, large city	894/3702 24.1 %	1111/3393 32.7 %	634/1925 32.9 %	1535/4109 37.4 %	1714/3662 46.8 %		0.026***
Small city	447/2038 21.9 %	539/2554 21.1 %	383/1474 26.0 %	1084/3185 34.0 %	936/2459 38.1 %		0.023***
Town	166/1063 15.6 %	140/913 15.3 %	87/508 17.1 %	196/732 26.8 %	238/685 34.7 %		0.034***
Countryside	376/7967 4.7 %	537/8699 6.2 %	517/5249 9.8 %	1722/11436 15.1 %	1853/9717 19.1 %	3324/14135 23.5 %	0.040***
Urban (2014)						3483/7621 45.7 %	
Number of children born							
Ε^2^ across categories of variable	0.040***	0.032***	0.028***	0.030***	0.029***	0.022***	
0	216/1388 15.6 %	213/1350 15.8 %	161/876 18.4 %	495/1857 26.7 %	598/1694 35.3 %	730/1881 38.8 %	0.045***
1	329/1797 18.3 %	383/2037 18.8 %	314/1281 24.5 %	821/2793 29.4 %	891/2544 35.0 %	1118/3097 36.1 %	0.025***
2	499/2400 20.8 %	668/2822 23.7 %	434/1815 23.9 %	1260/4060 31.0 %	1257/3586 35.1 %	2009/5422 37.1 %	0.017***
3	423/2411 17.5 %	512/2723 18.8 %	369/1808 20.4 %	989/3957 25.0 %	1071/3588 29.8 %	1658/5300 31.3 %	0.015***
4	226/1922 11.8 %	261/2072 12.6 %	177/1196 14.8 %	503/2610 19.3 %	504/2227 22.6 %	786/3242 24.2 %	0.016***
5 or more	189/4851 3.9 %	288/4553 6.3 %	164/2177 7.5 %	468/4185 11.2 %	422/2886 14.6 %	507/2815 18.0 %	0.027***
Wealth index							
Ε^2^ across categories of variable	0.081***	0.043***	0.024***	0.023***	0.014***	0.003***	
Low	71/2511 2.8 %	168/1533 11.0 %	109/729 15.0 %	329/1370 24.0 %	266/988 26.9 %	302/851 35.5 %	0.096***
Middle	366/6276 5.8 %	335/5620 6.0 %	202/2421 8.3 %	613/4911 12.5 %	1019/4922 20.7 %	4315/14619 29.5 %	0.072***
High	1445/5982 24.2 %	1823/8405 21.7 %	1309/6005 21.8 %	3595/13181 27.3 %	3457/10614 32.6 %	2190/6286 34.8 %	0.012***
Literacy							
Ε^2^ across categories of variable	0.142***	0.103***	0.087***	0.065***	0.072***	0.040***	
Illiterate	214/7896 2.7 %	343/7749 4.4 %	239/4015 6.0 %	891/7902 11.3 %	778/5823 13.4 %	964/5815 16.6 %	0.032***
Partly literate	95/1506 6.3 %	129/1248 10.3 %	85/685 12.4 %	195/1208 16.1 %	252/1175 21.4 %	262/1054 24.9 %	0.034***
Fully literate	1573/5364 29.3 %	1853/6557 28.3 %	1295/4448 29.1 %	3438/10294 33.4 %	3711/9514 39.0 %	5578/14880 37.5 %	0.007***

The totals do not always add up to *N* = 97,274 due to missing values on the independent variables

Significance: *: 0.050; **: 0.010; ***: 0.001

The results show that the increasing opposition to FGM cannot be attributed completely to the entry of new cohorts in the study population. Opposition to FGM increased significantly among all birth cohorts over the period studied. Furthermore, although opposition to FGM varies somewhat by birth cohort (except for 2003), the very small proportion of variance explained (E^2^ < 0.005) implies that this variation is small.

Religion has a clear effect on the belief that FGM should be stopped. The results in Table 1 show that ever married Christian women in Egypt have always expressed more opposition to FGM than their Muslim counterparts, but also that opposition spread faster among Christians than among Muslims. Among Christians, opposition to FGM increased from 39.0 % in 1995 to 76.1 % in 2014, while for Muslims the corresponding percentages are 11.3 and 29.5 %, respectively. The largest difference in opposition to FGM is observed between women who were cut themselves and those who were not. Among uncircumcised women, there is very strong opposition to FGM, with between 75.1 and 89.5 % believing that the practice should be discontinued, depending on the survey year. Among cut women there is significantly less opposition to FGM, but even among this group the percentage who believe that FGM should be discontinued increased steadily from 10.4 % in 1995 to 27.3 % in 2014. Women with many children are also less likely to oppose FGM, but even among those with 5 or more children opposition to FGM rose from 3.9 % in 1995 to 18.0 % in 2014. Among women with no or few children, about 37 % believe the practice of FGM should be stopped.

Opposition to FGM increased among all occupational groups, albeit not to the same degree (see Table 1). The only exception consists of women who work as unskilled manual workers, among whom there was no change in opposition to FGM. Among professional, technical and managerial workers and among clerical workers, opposition to FGM was always relatively strong and further increased between 1995 and 2014. A strong increase in opposition to FGM is observed among women who work in sales and in services and among skilled manual workers. These three occupational groups all had relatively low levels of opposition to FGM back in 1995, but experienced more rapid increases in opposition to FGM, at least up to 2008, and a decline by 2014. The occupational categories where ever married women’s opposition to FGM spread the least are agriculture and unskilled manual labor, but even among those working in agriculture a substantial increase in the opposition to FGM was observed. Up to 2008, women from wealthy households (score 7–8) were more likely than those from poorer households to oppose FGM. However, by 2014 opposition to FGM is similar among women from poor and wealthy households. Between 1995 and 2014, opposition to FGM increased significantly in all wealth categories.

Throughout the survey period, opposition to FGM is always strongest among women with higher levels of education. As shown in Table 1, opposition to FGM increased substantially among all educational categories, and the higher the educational level of a woman, the more likely she is to oppose FGM. The only exception pertains to women with higher (tertiary) education. This group of women already expressed strong opposition to FGM in 1995, and no clear trend is observed between 1995 and 2014. Trends in opposition to FGM by partner’s level of education show similar results.

Table 1 further confirms that opposition to FGM is strongest in larger cities and weakest in the countryside. Opposition to FGM also spread slower among countryside residents. In the capital and other large cities the percentage of women who oppose FGM increased from 24.1 % in 1995 to 46.8 % in 2008, but among women in the countryside the percentage increased from 4.7 % in 1995 to only 19.1 % in 2008, and 23.5 % in 2014. The results for the various geographical regions further confirm this observation. Opposition to FGM is most widespread in the Urban Governorates, where it increased from 25.2 % in 1995 to 52.4 % in 2014. In rural Upper Egypt there is less opposition to FGM, with an increase from only 4.2 % in 1995 to 19.5 % in 2014.

The results in Table 1 also suggest that the spread of FGM opposition slowed down between 2008 and 2014 among nearly all status categories. In some categories, opposition even decreased, most notably among those that already expressed strong opposition to FGM in 2008, such as respondents with professional, technical or managerial occupations, those who work in sales, services or skilled manual occupations, who have higher education or havea partner with higher education, and who are fully literate. It is also worth noticing that the explanatory power of many of the status variables (Ε^2^ across categories of variable) declined substantially over time. For example, this is the case for FGM status, labor market status, educational level, partner’s educational level, number of children ever born, wealth index, and literacy. This decline in explanatory power over time implies that these characteristics become less important for distinguishing between those who oppose FGM and those who do not. It further indicates that opposition to FGM is no longer confined to well-defined segments of Egyptian society. Indeed, the results in Table 1 show that the belief that FGM should be stopped increased among all status categories, and that it diffused among the general population.

**Fig. 2:**
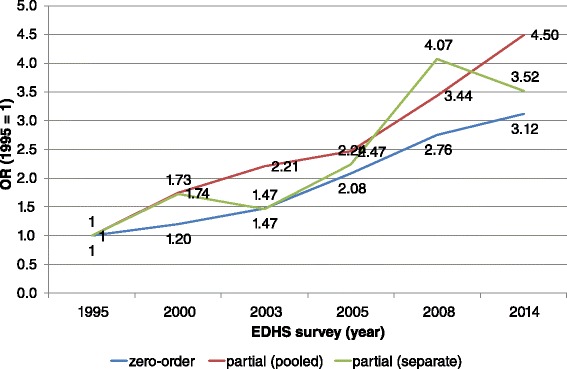
Observed and partial odds ratios for believing FGM should be stopped, by EDHS survey (ref: EDHS 1995. OR = 1)

Figure 2 assesses the extent to which the observed trends in opposition to FGM can be attributed to changes in the socioeconomic status of ever-married women. The figure shows three trend lines of the relative odds or odds-ratios (OR) of favoring the discontinuation of FGM, compared to the 1995 level. The first line (labelled “zero-order”) shows trends in the unadjusted relative odds of opposing FGM, which is identical to the prevalence rates shown in Fig. 1. Each of the lines shows the estimated change in the odds of favoring the discontinuation versus the continuation of FGM compared to the odds in the 1995 EDHS (OR = 1). The second line (labelled “partial (pooled)”) shows trends in the relative odds of opposing FGM after controlling for the status variables in the pooled sample, which assumes that the effects of the status variable remain constant over time. The third line shows trends in the relative odds of opposing FGM after controlling for the same status variables using the un-pooled (separate) datasets, which allows the effect of the status variables to vary over time.

The unadjusted ORs show that opposition to FGM steadily increased over the period studied to an OR of 3.12 in 2014. All zero-order ORs are significant at *p* < 0.001. Upon controlling for the status variables in the pooled analysis, the overall trend in opposition to FGM remains upward, but only in 2005, 2008 and 2014 is there significantly higher opposition than in 1995 (all three survey years were significant at *p* < 0.001). When controlling for the effects of the control variables using the separate analyses (which allows the effect of the control variables to vary over time), the trend become somewhat erratic, but overall opposition to FGM still increases. Again only the 2005 (*p* < 0.050), 2008 (*p* < 0.001) and 2014 (*p* < 0.010) ORs are significant. These results suggest that the observed trends in FGM opposition cannot simply be attributed to changes in the socio-demographic composition of the Egyptian population, such as an increase in women’s education or urbanization. It further confirms the findings from Table 1 which indicate that opposition to FGM is increasing among all segments of Egyptian society.

**Fig. 3:**
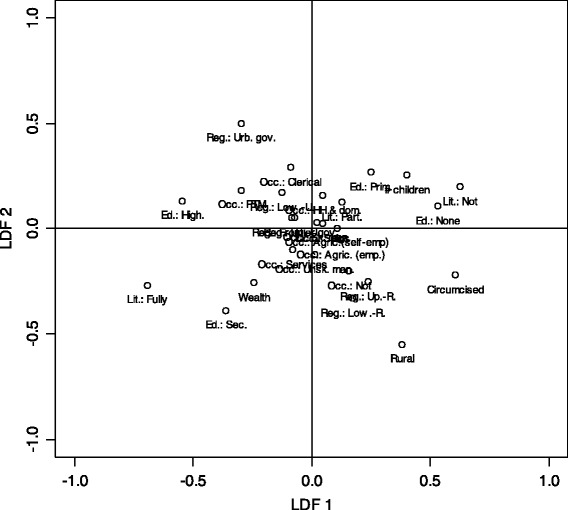
Discriminant analysis: Correlation of items with linear discriminant functions (LDF). Lit: Literacy (Not: Illiterate, Part.: Partially literate, Fully: Fully literate); LMS: Labor market status (Not: Not working, PTM: Professional, technical and manager occupations, Clerical: Clerical occupations, Sales: Sales occupation, Agric. (self-empl.): Agriculture (self-employed), Agric. (emp): Agriculture (employee), HH & dom: Household and domestic occupations, Services: Service occupations, Sk. man.: Skilled manual, Unsk. man.: Unskilled manual); Circumcised: Is respondent circumcised; # children: Total number of children born; Rural: Rural residence; Reg,: Region (Urb. Gov.: Urban governorates, Low.-R: Lower Egypt – Rural, Low.-U: Lower Egypt – Urban, Up.-R: Upper Egypt – Rural, Up.-U: Upper Egypt – Urban, Frontier. Gov.: Frontier governorates); Ed: Education level of respondent (None: No formal education, Prim.: Primary education, Sec.: Secondary education, High.: Higher education); Wealth: Wealth index

To summarize all these findings a discriminant analysis is run on the pooled dataset. Discriminant analysis estimates linear functions that best account for the multivariate inter-group variation. For this analysis, the groups are defined by combining the EDHS survey wave and the respondent’s attitude toward the discontinuation of FGM, resulting in 12 groups (6 survey waves × 2 attitudes). Two linear discriminant functions are retained that together accounted for 83.3 % of the inter-group variation. The first discriminant function (LDF1) accounts for 61.6 % of the between-group variance on the variables included in the analysis and the second (LDF2) for an additional 21.7 %. Figure 3 displays the correlations between the status categories and the two LDFs. Focusing only on the strong correlations (|*r*| > 0.30) shows that respondents who score high on the first LDF are more likely to be uneducated, illiterate, uncircumcised, to have many children, and to live in a rural area; while those who score low are more likely to have secondary or higher education or to be fully literate. The second LDF is orthogonal to the first one, which implies that it only captures differences in the variables or inter-group variation that are not accounted for by the first LDF. A higher score on the second LDF correlates positively with residing in the Urban Governorates, and negatively with living in the countryside or rural Lower Egypt, and having secondary education. Thus the first LDF can be described as a modern (low score) versus traditional (high score) axis, while the second LDF is a residual urban vs. rural axis, as respondents scoring low on this axis tend to be people living in rural areas with a secondary education.

**Fig. 4:**
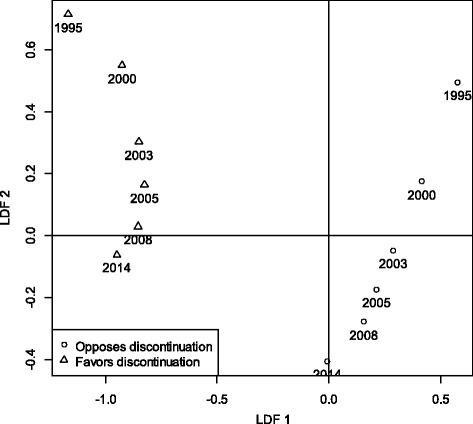
Discriminant analysis, mean (centroid) scores for women who believe FGC should be discontinued and women who do not believe it needs to be discontinued, by EDHS survey

Figure 4 shows the mean (centroid) scores for the 12 groups on the two LDFs. The first LDF clearly discriminates between supporters and opponents of FGM. The second dimension discriminates between the different EDHS waves and captures a time dimension and, to a much lesser extent, the differences between supporters and opponents of FGM. Table 2 shows a hierarchical decomposition (Type I Sum of Squares) of the variation of both LDFs. The variation accounted for by the EDHS waves is extracted first, followed by the group’s attitude toward FGM, and finally the interaction between the two. The 12 groups account for 20.9 % of the variance of LDF 1, 16.5 % of which (or 79 % of the total variance accounted for) can be attributed to the attitude toward FGM. For LDF 2, the 12 groups account for only 9.0 % of the variance, with the group’s attitude toward FGM contributing only 1/6^th^ of this total effect.

**Table 2:** Hierarchical decomposition of the variance of the LDFs

% of variance on LDF accounted for by	LDF 1	LDF 2
DHS wave	3.7 %	7.4 %
Attitude towards FGM	16.5 %	1.5 %
Interaction	0.8 %	0.0 %
Total	20.9 %	9.0 %

All effects are significant at *p* < 0.001

As shown in Fig. 4, on LDF 1 (the modern vs. traditional axis) all the groups who believe FGM should be discontinued score significantly lower (more modern) than those who do not (more traditional). But at the same time we observe a significant trend toward the center for both supporters and opponents of FGM. For women who believe FGM should be discontinued, the average score on LDF 1 increased until 2005, which means that opposition to FGM was spreading into more traditional segments of society. In subsequent surveys the scores decrease until 2014, when they reach a level similar to that observed for the year 2000. Among women who do not oppose FGM, the mean scores on LDF1 decrease over time.

The shift over time is substantially larger for women who support FGM than for those who oppose it. For women who support FGM the maximum shift is 0.581; for women who want FGM to be discontinued it is only 0.342 (not shown). This indicates that while, on average, supporters of FGM became more modern over time due to social changes such as improved educational opportunities and urbanization. Nevertheless, the supporters of FGM always remain substantially more traditional than the opponents. As anti-FGM attitudes also start to spread into more ‘traditional’ segments of society, the opponents of FGM become, on the average, less modern and more traditional. The differences in LDF1 scores between the supporters and opponents of FGM also tended to become smaller over time. While in 1995 the gap between the two centroid scores was still 1.74, by 2014 it had narrowed to 0.94 (not shown). All these findings point to three observations: first, the difference between the two groups reduced over time; second, although opposition to FGM was originally concentrated among the well-educated urban segments of Egyptian society, it has become more widespread in all segments of society; and third, the group of women who are support FGM is also modernizing rapidly as the overall level of education of Egyptian women is increasing, although on average they remain significantly more traditional than women who believe FGM should be discontinued.

Since the second LDF (the residual rural vs. urban axis) is orthogonal to the first it only captures differences that are not yet captured by the first LDF. At each survey wave, women who want to discontinue FGM score significantly higher (at *p* < 0.001) than women who support FGM on this second LDF, indicating that the former group contains women that are more likely to live in urban areas than predicted by the first LDF, i.e., are women who are on other characteristics more traditional but live in urban areas, than supporters of FGM. For both groups, however, there is substantial shift over the period studied towards the negative end of the axis, i.e., with respondents who are more likely to live in rural areas and to have secondary education than predicted by the first LDF.

## Discussion

FGM is deeply embedded in Egyptian society and tradition. At present, the overwhelming majority of girls are still being cut. All evidence, including this study, shows that support for the practice has remained strong in almost all segments of Egyptian society. But there are some indications that support for FGM may be waning. For instance, the EDHS data (see Fig. 1) show that the percentage of ever-married women who believe FGM should be discontinued increased from 12.7 % in 1995 to 31.3 % in 2014. Other studies also show a decline in support for or in the prevalence of FGM in Egypt [3, 5–7, 37].

This study confirms that opposition to FGM originated among the more modernized segments of Egyptian society, i.e., urban, better-off and highly educated women, as well as among the small minority of non-circumcised women. These findings are consistent with the literature that shows opposition to FGM is most common among better educated urban residents [8, 30, 32, 38]. Although these groups continue to oppose FGM, opposition is spreading to other segments of society, including more disadvantaged and traditional groups. While the growth of the more modern segments of society (e.g., increases in the percentage of educated women) contributed to the changes in attitudes toward FGM, it is only one part of the story. Women’s desire to abolish FGM has also been diffusing to groups of women who used to cling to more traditional values. The findings here are consistent with theories about innovation diffusion and influence [28, 29, 33]. Some groups in society do function as opinion leaders and reference groups for others.

FGM is entrenched in community traditions and structures, and often there are strong social pressures to conform to tradition. In many cases it would be considered poor parenting not to have one’s daughters cut, as this would diminish the social status not only of these daughters but of the entire family [22, 23, 30, 37]. Often these community structures and processes not only limit exposure to new information, including messages that oppose FGM, but also limit their ability to act on them. Groups that have weak traditional community social controls are more likely to be responsive to messages that discourage FGM, and more likely to change their attitudes toward FGM and to stop practicing it. The two segments of society that are known to be least susceptible to traditional pressures are the modern elites and marginal groups (such as the very poor, and religious and ethnic minorities). Because of the social barriers that marginal groups tend to face, they are less likely to function as role models. The modern elites, on the other hand, are much better situated to act as role models. Members of modern elites are often urban, better educated, employed in the more modern sectors of the economy, and exposed to foreign media and ideas. Moreover, because these groups rely less on traditional sources of status, they are better equipped to abandon traditional practices. These elites can increase or maintain their status and ensure their daughters’ future by means other than FGM. Therefore, it is less important for them to have their daughters cut.

A main limitation of the results presented here is that the EDHS samples do not include never-married women. Therefore, the younger cohorts (15–19 and 20–24) in the samples may not be representative of all women in those age categories. As early marriage is more common among more traditional segments of society the EDHS may underestimate the opposition to FGM in these age groups [5]. The current analysis does not provide any information about opposition to FGM among men. However, although the EDHS surveys are limited to ever married women, the findings may be indicative of trends in the larger population.

A second limitation of this study is that it focuses exclusively on attitudes towards FGM. Although attitudes feature prominently in many models of behavior change [9, 11, 12], changing attitudes toward FGM is not sufficient to eliminate the practice of FGM. Even if women are ready and willing to stop cutting their daughters, they also need to be able to do so [13, 14]. Whether or not to have one’s daughter undergo FGM is rarely an individual decision by the mother. In most cases such decisions involve other household and family members. Hence, it remains unclear to what extent the observed changes in attitudes towards FGM among ever-married women will result in an actual decline in the prevalence of daughters who experience FGM.

## Conclusion

The results from this study confirm that opposition to FGM is spreading among Egyptian women. To the extent that the spread of these sentiments is due to the improvement of the position and status of women in Egyptian society and mainly driven by improvements in education there may not be a need for additional campaigns that specifically discourage FGM. Instead, it may be better to focus on improving Egyptian women’s position in general, for example by expanding educational opportunities for women. Although we observe that women’s opposition to FGM has been spreading beyond these modernized and, to some extent. Westernized groups, substantial differences remain among the various segments of society, with some groups of women still lagging considerably. For these groups additional intervention campaigns may remain necessary to discourage FGM.

Our analyses do not allow us to evaluate the effect of the legal ban of FGM on societal attitudes towards FGM. Although one can hope that the legal ban on FGM will speed up its delegitimation, as has been the case with bans on other harmful practices, it remains unclear whether this has actually been the case. We observe that FGM opposition kept spreading after the ban went into effect, although at a slower pace. This finding is consistent with the arguments of Hassanin and Shaaban [37] who also concluded that the impact of the legal ban on the prevalence of and attitudes towards FGM was limited at best. Of course, much depends on how rigorous the new laws will be enforced. In the past the Egyptian authorities hardly ever enforced measures against FGM, as influential parts of society opposed these measures. To be successful such a ban needs the support of influential groups in society, including the leading clergy. In this regard the political events of the past few years in Egypt certainly do not bode well for the fight against FGM. There is little doubt that the fight against FGM has not been a priority in recent years, and currently FGM does not feature prominently on the government’s agenda. Thus, it appears that efforts to eradicate FGM in Egypt may be stalling, only a small number of cases have been brought to justice since the new law went into effect in 2008 [39]. The resurgence of more traditional forms of Islam may mean that attitudes toward FGM may become less determined by structural factors, such as the socio-demographic characteristics included in this study. Nevertheless, comparison of the 2008 and 2014 data shows that sentiments opposing FGM have increased among almost all segments of Egyptian society, although the increase in opposition to FGM has slowed down compared to earlier this century. Whether this is due to the events of the last few years, and whether the increase in opposition to FGM will continue remains to be seen. However, the current political, cultural and economic situation in Egypt does not appear favorable for the social advancement of women, and therefore for the battle against FGM.

## Abbreviations

DHS: Demographic and Health Survey
EDHS: Egypt Demographic and Health Survey
IRB: Institutional Review Board
FGM: female genital mutilation
HSD: honestly significant difference
LDF: linear discriminant function

## Footnotes

1 The answer option “other” was only included in the 2000 and 2003 surveys.

2 Although the DHS data include a wealth index, this indicator measures relative wealth within the country at the time of the survey. As such, the scores of this wealth index cannot be compared across countries or over time [40, 41].

3 The DHS classifies urban areas into large cities (capital cities and cities with over 1 million population), small cities (population over 50,000), and towns (other urban areas). All rural areas are assumed to be countryside. Note that the 2014 survey only distinguishes between rural and urban locations.

4 Including these variables would have caused these categories to very strongly discriminate between the waves that included them and those that did not.
